# Physiolometrics and the puzzle of methodical acumen

**DOI:** 10.1113/EP091406

**Published:** 2023-08-09

**Authors:** Jacob Peter Hartmann, Markus Harboe Olsen, George Rose, Damian M. Bailey, Ronan M. G. Berg

**Affiliations:** ^1^ Centre for Physical Activity Research Copenhagen University Hospital – Rigshospitalet Copenhagen Denmark; ^2^ Department of Clinical Physiology and Nuclear Medicine Copenhagen University Hospital – Rigshospitalet Copenhagen Denmark; ^3^ Department of Neuroanaesthesia The Neuroscience Centre Copenhagen University Hospital – Rigshospitalet Copenhagen Denmark; ^4^ Neurovascular Research Laboratory Faculty of Life Sciences and Education University of South Wales Pontypridd UK; ^5^ Department of Biomedical Sciences Faculty of Health and Medical Sciences University of Copenhagen Copenhagen Denmark

‘It is of the highest importance in the art of detection to be able to recognize, out of a number of facts, which are incidental and which vital!’ These words, spoken by Sherlock Holmes in *The Adventure of the Reigate Squire* (later also known as *The Adventure of the Reigate Puzzle*) published in the June issue of *The Strand Magazine* in 1893, resonate with our quest as physiologists for methodological rigor to this day. It is tempting to speculate that John Newport Langley (1852−1925), who during that period assumed control of *The Journal of Physiology* and became its most notorious Editor‐in‐Chief to date (Bailey et al., 2023), much like his contemporaries in The Physiological Society, found inspiration in the unwavering pursuit of deductive excellence exemplified by Sherlock Holmes.

At the turn of the century, Langley's insistence on a meticulously structured approach to the analysis and presentation of data fundamentally altered physiology as an experimental science, and continues to do so to this day (Bailey et al., 2023). During those years, many prevailing theories such as the unitary view of brain function (i.e., absence of localised function), the theory of urine secretion in the kidney, and the concept of oxygen secretion in the lungs, to mention a few, were revisited. One by one, they were debunked by uncovering a flawed empirical basis due to methodological limitations or the development of more accurate experimental models and measurement techniques to help advance understanding (Krogh, 1910; Langley & Sherrington, 1884; Starling, 1899). Indeed, the discipline focused on the advancement and systematic evaluation of physiological measurement techniques, which we refer to as *physiolometrics*, is essential to modern physiology. Here, we will briefly define the main physiolometric concepts of validity and reliability and describe how they may be used to systematically evaluate both existing and new physiological methods.

*Validity* pertains to the ability of a method to accurately reflect the true physiological variable. In physiolometrics, it mainly encompasses three domains: logical validity, construct validity and criterion validity (George et al., 2000). *Logical validity* entails a qualitative evaluation of whether the measurement method, principles and assumptions align with providing meaningful measurements, based on established principles of physics, biochemistry, biology and physiology. However, while an assessment of logical validity can be used to infer whether the theoretical basis and measurement principle of a method is fundamentally flawed, it does not provide any quantitative measure of a method's validity.

In contrast to logical validity, construct and criterion validity are based on quantitative statistics‐based assessments. These quantitative measures include sensitivity and specificity of the method for detecting a given change in a physiological parameter, and an assessment of systematic error – measurement bias. Measurement bias is defined as predictable deviations from the true value, arising from flaws or biases in the measurement process, such as faulty equipment, measurement instrument calibration issues, or biased measurement procedures. *Construct validity* examines how well a measurement (the ‘construct’) behaves in response to various physiological stimuli and in relation to other physiological parameters. Construct validity can be evaluated using simple paired tests to determine whether relevant changes in the measure to a given intervention occurred simply by chance alone, or for example, by correlational analyses to assess whether the measure behaves as expected in relation to other variables (Cronbach & Meehl, 1955). Discriminant validity is a subdomain of construct validity that explores the measure's ability to distinguish between individuals expected to differ. In the case of measures that have established thresholds, sensitivity and specificity can be employed, categorizing the measure as either normal or abnormal. For continuous measures without established thresholds, construct validity can be investigated using receiver operating characteristic analysis and calculating the area under the curve (Olsen et al., 2022).

*Criterion validity* involves the comparison of a given method to an established reference (or criterion) method. Importantly, the formal assessment of criterion validity should not rely on simple difference tests, as this approach obscures individual variability, or on correlation techniques as these are not suitable for repeated assessments of the same variable, and do not account for systematic error. The most suitable approach for assessing criterion validity is arguably the Bland–Altman plot, where the difference between each measurement obtained by the two methods is plotted against the mean of the two with the latter considered to represent the best estimate of the ‘true’ value (Bland & Altman, 1986; Bunce, 2009). This offers direct estimates of systematic error (bias) between the methods using the ‘limits of agreement’ which essentially represent a range of validity, given that the data do not demonstrate heteroscedasticity, that is, that the magnitude of error does not increase with higher measured values. A complementary approach is the use of the intraclass correlation coefficient (ICC), but this does have considerable limitations and pitfalls as outlined below in relation to the assessment of reliability. Furthermore, it must be noted that neither Bland–Altman plots nor ICCs provide any clues as to which method is superior when there is evidence of substantial systematic error. In such cases, it may be necessary to assess how the measure of interest obtained by the two methods predicts a given outcome.

*Reliability* focuses on the consistency of measurement and aims to quantify both inherent and random errors of the measurement and encompasses aspects such as test–retest reliability and inter‐rater reliability. Furthermore, reliability pertains to either the consistency of measurement when repeated under identical experimental conditions (repeatability) or its consistency across varying experimental conditions (reproducibility) (Bartlett & Frost, 2008). Random error refers to unpredictable fluctuations or variations in measurement that occur randomly and without any consistent pattern. It is typically caused by chance factors, such as human error, natural variability, or environmental influences that affect the measurement process. As for criterion validity, the use of Pearson's correlation to assess reliability can create an exaggerated impression of reliability and should be avoided. Paired tests between the mean values in the case of test–retest data do not directly inform reliability, since any random error will add to the variability within and between the repeated measure, and thus increase rather than decrease the *P*‐value. Thus, paired tests should only be used as a ‘gatekeeper method’ to ensure internal consistency of the data, that is, to rule out systematic errors at the group level that will render the given dataset inappropriate for test–retest reliability assessment (Silbernagel et al., 2005). Reliability can be evaluated in terms of absolute and relative reliability, where absolute reliability focuses on the magnitude or extent of measurement error quantified in the same unit as the measure of interest, whereas relative reliability provides information about the relative contribution of measurement error to the overall variation in the measure and is usually provided as a percentage.

Absolute reliability can be assessed through a Bland–Altman plot as outlined above, as well as by calculation of the closely related smallest real difference (SRD). SRD is easily interpretable because it provides an estimate of the maximal difference there will be between two measurements on 95% of occasions (Vaz et al., 2013).

A commonly used relative reliability estimate is the coefficient of variation (CV) reflecting the relationship between the standard deviation within the group (also known as the standard error of measurement) and the mean (Searls, 1964), and in reliability studies it will furthermore often be relevant to calculate 95% confidence intervals of the CV, which is possible by a simulation procedure based on the distribution of the estimates of mean and residual variance from a linear mixed model (Liu, 2012). Caution should, however, be exercised when the mean is close to zero, as it can lead to inaccurate results (e.g., infinite CV regardless of standard error of measurement). Furthermore, if the CV is reported in studies measuring graded steps, as often done in exercise physiology, the CV will often decrease as intensity increases, because the mean value of the estimate increases relatively more than the standard error of measurement. As for many other physiolometrics, the cut‐off values for acceptable and unacceptable CVs are somewhat arbitrary and depend on the research field.

The ICC, which encompasses several subtypes depending on scope and type of data, is another widely used measure for relative reliability and can conveniently be used to classify the reliability of a method categorically as poor, moderate, good or excellent (Koo & Li, 2016). However, the ICC should be reported and interpreted cautiously due to its sensitivity to variations within and between groups (Madsen et al., 2023). This basically means that if the ICC is reported on a very heterogenous population, a high within‐group standard deviation may lead to a high ICC no matter how flawed the method is. Hence, the ICC alone does not offer a comprehensive assessment of reliability and should not be considered independently of other reliability measures. A more rarely used reliability measure is the root mean square deviation coefficient, which provides a measure of the overall deviation (or error) between the observed and predicted values. It can also be applied for the test–retest study set‐up, but one should be cautious because the measure is very sensitive to outliers and does not differentiate between random and systematic error (Barchard, 2012). For the assessment of absolute and relative reliability, we have recently developed a publicly available *calcrel* function in the *clintools* package in R (https://cran.r‐project.org/web/packages/clintools/index.html).

When considering (relative) reliability, physiological biomarkers are dynamic metrics subject to natural variation. This variation encompasses both analytical and, more prominently, biological components that collectively contribute to what is known as the *critical difference* (Fraser & Fogarty, 1989). This describes random variation around a homeostatic point, indicative of the change that must occur before a true physiologically or clinically relevant difference can be claimed. While this concept emanates from the field of clinical biochemistry applied to metabolic biomarkers of exercise stress (Davison et al., 2012), it is less well known in clinical and other fields of physiology where it can optimise the interpretation and stratification of responses (Rose et al., 2018). Furthermore, the critical difference may be an important consideration in interventional studies with responder analyses where claimed ‘responders’ may simply be artefacts within a defined zone of natural variation.

As was the case 130 years ago when Langley may have read the works of Sir Arthur Conan Doyle, the distinction between incidental and vital facts, that is, between measurement error and the true underlying value, remains a defining feature of physiology as an experimental science. So, for us as physiologists to appropriately design and conduct studies and provide results that uncover the fundamental mechanisms of life, both in the healthy and diseased state, we need to embrace physiolometrics. This will help ensure that rather than being discarded due to methodological and/or statistical flaws, our findings may remain relevant for future generations of physiologists, even as they develop and overturn today's major concepts and theories as part of the self‐correcting nature of science (Whipp, 2010). Thus, the systematic and critical assessment of the validity and reliability of our methods is a *sine qua non* if our results are to stand the test of time. Or as Sherlock Holmes would put it himself: ‘It is elementary!’

## AUTHOR CONTRIBUTIONS

All authors have read and approved the final version of this manuscript and agree to be accountable for all aspects of the work in ensuring that questions related to the accuracy or integrity of any part of the work are appropriately investigated and resolved. All persons designated as authors qualify for authorship, and all those who qualify for authorship are listed.

### FUNDING INFORMATION

The Centre for Physical Activity Research is supported by TrygFonden Grants ID 101390, ID 20045, and ID 125132. J.P.H. is funded by HelseFonden and Rigshospitalet. D.M.B. has received funding from a Royal Society Wolfson Research Fellowship (#WM170007) and the Higher Education Funding Council for Wales.

## CONFLICT OF INTEREST

None of the authors have any competing interests to disclose.
